# A Comparison of Diets Supplemented with a Feed Additive Containing Organic Acids, Cinnamaldehyde and a Permeabilizing Complex, or Zinc Oxide, on Post-Weaning Diarrhoea, Selected Bacterial Populations, Blood Measures and Performance in Weaned Pigs Experimentally Infected with Enterotoxigenic *E. coli* †

**DOI:** 10.3390/ani5040403

**Published:** 2015-11-23

**Authors:** Ingunn Stensland, Jae Cheol Kim, Bethany Bowring, Alison M. Collins, Josephine P. Mansfield, John R. Pluske

**Affiliations:** 1 School of Veterinary and Life Sciences, Murdoch University, Murdoch, WA 6150, Australia; E-Mails: ingunnstensland@gmail.com (I.S.); J.Mansfield@murdoch.edu.au (J.P.M.); 2 Pork Innovation, Department of Agriculture and Food Western Australia, South Perth, WA 6150, Australia; E-Mail: jae.kim@agric.wa.gov.au; 3 New South Wales Department of Primary Industries, Elizabeth Macarthur Agricultural Institute, PMB 4008, Narellan 2567, Australia; E-Mails: bethany.bowring@dpi.nsw.gov.au (B.B.); alison.collins@dpi.nsw.gov.au (A.M.C.)

**Keywords:** cinnamaldehyde, enterotoxigenic *E. coli*, organic acids, post-weaning diarrhoea, pigs, zinc oxide, permeabilizing complex

## Abstract

**Simple Summary:**

This experiment was conducted to assess the effects of three diets on diarrhoea, performance (weight change, feed intake and feed conversion ratio), selected bacterial populations and blood measures of weaner pigs infected with enterotoxigenic *E. coli*. The three diets were: base diet (no antimicrobial compounds), base diet containing zinc oxide, and base diet containing a feed additive (blend of organic acids, cinnamaldehyde and permeabilizing complex). Only feeding zinc oxide decreased diarrhoea, with zinc oxide-fed pigs performing better than base diet-fed pigs. Zinc oxide-fed pigs performed similarly to pigs fed the organic acids, cinnamaldehyde and permeabilizing complex. Significant interactions between treatment and day after weaning were found for some bacterial populations, although the implications of such findings require further examination.

**Abstract:**

The effects of feeding a diet supplemented with zinc oxide (ZnO) or a blend of organic acids, cinnamaldehyde and a permeabilizing complex (OACP) on post-weaning diarrhoea (PWD) and performance in pigs infected with enterotoxigenic *E. coli* (ETEC) were examined. Additionally, changes in selected bacterial populations and blood measures were assessed. A total of 72 pigs weaned at 22 d of age and weighing 7.2 ± 1.02 kg (mean ± SEM) was used. Treatments were: base diet (no antimicrobial compounds); base diet + 3 g ZnO/kg; base diet + 1.5 g OACP/kg. Dietary treatments started on the day of weaning and were fed *ad libitum* for 3 weeks. All pigs were infected with an F4 ETEC on d 4, 5 and 6 after weaning. The incidence of PWD was lower in pigs fed ZnO (*p* = 0.026). Overall, pigs fed ZnO grew faster (*p* = 0.013) and ate more (*p* = 0.004) than the base diet-fed pigs, with OACP-fed pigs performing the same (*p* > 0.05) as both the ZnO- and base diet-fed pigs. Feed conversion ratio was similar for all diets (*p* > 0.05). The percentage of *E. coli* with F4 fimbriae was affected a day by treatment interaction (*p* = 0.037), with more *E. coli* with F4 fimbriae found in pigs fed ZnO on d 11 (*p* = 0.011) compared to base diet-fed pigs. Only significant time effects (*p* < 0.05) occurred for blood measures. Under the conditions of this study, inclusion of OACP gave statistically similar production responses to pigs fed ZnO, however pigs fed ZnO had less PWD compared to OACP- and the base diet-fed pigs.

## 1. Introduction

Weaning is a stressful process for pigs and typically causes a growth check, but the stressors of weaning can also render pigs more susceptible to gastrointestinal tract (GIT) diseases and dysfunction [1,2]. Post weaning diarrhoea (PWD) is associated with the proliferation of enterotoxigenic β-haemolytic strains of *Escherichia coli* (*E. coli*) [3], and can be a major cause of economic loss in a herd [4]. The commensal bacteria in the intestine, including *Lactobacillus* spp., play important roles in preventing the colonization of pathogens through competitive exclusion and excretion of bacteriocins capable of bacterial lysis [5]. Antibiotics and (or) mineral compounds such as zinc oxide (ZnO) have traditionally been used to control PWD and GIT dysbiosis [6,7,8]. Health and consumer concerns related to the development of antimicrobial resistance [9,10,11], and concerns related to the accumulation of minerals such as zinc in the environment [6], have resulted in the development of alternative strategies to maintain health and performance in the post-weaning period.

Organic acids in weaner pig diets can control PWD and enhance growth performance [12,13]. Acetic, formic and propionic acids have a direct effect on Gram-negative bacteria, impeding the replication of deoxyribonucleic acid [14]. Formic acid has been found to decrease colonization of enterobacteria in the lower part of the GIT of pigs [15], increasing growth efficiency when supplemented to market pigs [16] and having a positive effect on controlling PWD in pigs [17]. Propionic acid has also displayed positive effects on controlling PWD and a general improvement in performance [17]. Furthermore, acetic acid, when fed in a combination with formic, phosphoric and citric acid, was found to have positive effects on performance in newly-weaned pigs [18].

Essential oils (EO) are phytochemicals acquired from plant material [19], and are the plants’ natural defence mechanism against predators and pathogens [20]. There is considerable interest in the antibacterial and (or) antiviral properties of some EO for application in the post-weaning period [21,22]. In this regard, cinnamaldehyde was found to decrease faecal *E. coli* concentrations, with no effect on the concentration of faecal lactobacilli, in weaned pigs [23].

Organic acids and EO have different modes of action to antimicrobial growth promoters (AGP), and it is therefore unlikely that one alone is going to be able to substitute AGP to control PWD. A combination of organic acid blends and EO would give a broader spectrum of activity as organic acids exert their activity in feed and the upper part of the GIT whilst EO exert their activity more in the distal part of the GIT [24]. Furthermore, Gram-negative bacteria such as *E. coli* possess a cell wall in addition to their cell membrane, which prevents compounds such as organic acids from entering the bacteria and destroying vital cellular functions. A permeabilizing complex has been found to disrupt this cell wall, making the bacteria more susceptible to these compounds and contributing to enhanced post-weaning growth [25].

The hypothesis tested in this experiment was that a post-weaning diet supplemented with a blend of organic acids (propionic, formic and acetic), cinnamaldehyde and a permeabilizing complex (OACP) will decrease the incidence of PWD in pigs and increase growth performance following infection with enterotoxigenic *E. coli*. This product was examined against a base diet without any antimicrobial compounds and a diet containing ZnO, which is still commonly used in the post-weaning period to mitigate disease and improve performance.

## 2. Experimental Section

This study was reviewed and approved by the Animal Ethics Committee of Murdoch University (R2631/14). Animals were handled according to the Australian Code of Practice for the Care and Use of Animals for Scientific Purposes [26].

### 2.1. Animals, Experimental Design, Diets and Housing

A total of 72 entire male pigs (Large White × Landrace) weaned at an average of 22 days (d) of age and weighing 7.2 ± 1.02 kg (mean ± SEM) was used. The pigs were obtained from a commercial pig farm (Yarloop, Western Australia) on the day of weaning and transported to an experimental facility at Murdoch University. Due to supply issues the pigs arrived in two batches 3 days apart, but were placed on test according to the same timeframe. On arrival, the pigs were weighed and faecal rectal swabs were taken and cultured for baseline presence of β-haemolytic *E. coli*. Pigs were randomly allocated to their experimental diet in 6 replicate pens of 4 pigs per pen according to a completely randomised block distribution and live weight (3 treatments × 6 replicate pens per treatment × 4 pigs per pen; *n* = 72).

Treatments comprised three different diets: a base diet without any antimicrobial compounds; the base diet with 3 g ZnO/kg added; and the base diet with 1.5 g OACP/kg (Biotronic Top 3^®^, Biomin Australia Pty Ltd., Carlingford, Australia) added. The base diet, comprised mainly of wheat, soybean meal, barley and whey, was formulated to meet the animals’ requirements according to National Research Council [27] (10.4 MJ NE/kg, 0.9 g standardised ileal digestible lysine/MJ DE). Diet compositions and analysed gross energy and nutrient contents are presented in Table 1. The diets, along with water, were offered on an ad libitum basis for 3 weeks after weaning.

**Table 1:** Composition of experimental diets (g/kg, as fed basis).

Ingredient	Base Diet	ZnO	OACP
Barley	100.0	100.0	100.0
Wheat	492.2	489.2	490.7
Soybean meal	150.0	150.0	150.0
Blood meal	20.0	20.0	20.0
Fish meal	84.1	84.1	84.1
Whey powder	100.0	100.0	100.0
Canola Oil	34.2	34.2	34.2
L-lysine	2.71	2.71	2.71
DL-methionine	2.30	2.30	2.30
L-threonine	1.30	1.30	1.30
L-tryptophan	0.13	0.13	0.13
Vitamin/Mineral premix **^†^**	1.0	1.0	1.0
Limestone	5.2	5.2	5.2
Dicalcium phosphate	4.4	4.4	4.4
Salt (NaCl)	2.0	2.0	2.0
Zinc Oxide	0.0	3.0	0.0
Choline chloride (60%)	0.4	0.4	0.4
Biotronic Top 3^®^ **^‡^**	0.0	0.0	1.5
**Calculated composition**			
NE, MJ/kg	10.4	10.4	10.4
Protein	213	213	213
Fat	54	54	54
NDF	95	95	95
ADF	28	28	28
Calcium	9.0	9.0	9.0
Digestible phosphorus	4.5	4.5	4.5
Total lysine	14.1	14.1	14.1
SID lysine	13.5	13.5	13.5
SID meth + cysteine	8.1	8.1	8.1
SID threonine	8.5	8.5	8.5
SID tryptophan	2.4	2.4	2.4
SID isoleucine	7.7	7.7	7.7
SID leucine	14.8	14.7	14.8
**Analyzed composition**			
Dry matter	927	926	926
Gross energy, MJ/kg	17.6	17.3	17.6
Protein	221	218	224
Crude fibre	20	20	18
NDF	104	90	90
ADF	34	33	31
Zinc	0.22	2.55	0.22
pH	6.26	6.63	6.20

**^†^** Provided the following nutrients (per kg of air-dried diet): vitamins: A, 7000 IU; D3, 1400 IU; E, 20 mg; K, 1 mg; thiamine, 1 mg; riboflavin, 3 mg; pyridoxine, 1.5 mg; cyanocobalamin, 15 μg; calcium pantothenate, 10.7 mg; folic acid, 0.2 mg; niacin, 12 mg; biotin, 30 μg. Minerals: Co, 0.2 mg (as cobalt sulfate); Cu, 10 mg (as copper sulfate); iodine, 0.5 mg (as potassium iodine); iron, 60 mg (as ferrous sulfate); Mn, 40 mg (as manganous oxide); Se, 0.3 mg (as sodium selenite); Zn, 100 mg (as zinc oxide); BJ Grower 1, BioJohn Pty Ltd., Perth, Western Australia; **^‡^** Biotronic Top 3^®^, Biomin Australia Pty Ltd., Carlingford, Australia (a mixture of an organic acid blend, cinnamaldehyde and permeabilizing complex (OACP)). NDF, neutral detergent fibre; ADF, acid detergent fibre; SID, standardised ileal digestible.

Pigs were kept in pens of metal wire-meshed construction with plastic flooring and with a space allowance of at least 0.6 m^2^ per pig. Each pen was equipped with a nipple water drinker and a plastic feeding trough. Pens were in three different rooms, with 6 pens per room and 2 pens per treatment in each room. The ambient temperature was maintained at 26.3 ± 1.0 °C (mean ± SD). Pigs were monitored twice daily and weighed weekly. Feed disappearance from each pen was recorded weekly and feed wastage was visually assessed (by the same person) and recorded daily to calculate feed intake, body weight gain, and feed conversion ratio.

### 2.2. Induction of PWD with Enterotoxigenic E. coli and Measurements of PWD

Pigs were infected with an enterotoxigenic *E. coli* (ETEC, serotype O149:K98:K88; toxins LT, ST, STb, EAST) on d 4, 5 and 6 after weaning. Inoculation cultures of ETEC were prepared as described by Heo, *et al.* [28]. All pigs were orally dosed, using mild restraint, with the inoculum via a drench gun to provide 9 mL aliquots of 1.03 × 10^9^ colony forming units (CFU)/mL of ETEC per pig. Faecal swabs were taken on d 0, 3, 5, 7, 9 and 11 to assess faecal shedding of ETEC, by inserting a cotton swab into the anus. Swabs were inoculated on sheep blood (50 mL/L) agar plates (Path West Laboratories, Perth, Western Australia). Plates were incubated overnight at 37 °C and assessed based on morphology and haemolysis. Scores were given on a scale from 0 to 5 according to the number of streaked sections containing viable haemolytic *E. coli*, where 0 was no growth and 5 was growth out in the fifth section of the plate [28].

Faecal consistency and the incidence of diarrhoea of individual pigs were visually assessed daily, by the same person, for 21 d after weaning. A score between 1 and 4 was given, as follows: (1) firm, well-formed faeces; (2) soft formed faeces; (3) soft and loose shape; or (4) watery liquid consistency, with this considered as diarrhoea. To allow for statistical analysis the scores were converted into percentiles (1 = 0%, 2 = 33.3%, 3 = 66.7% and 4 = 100%). The diarrhoea index (DI) was calculated as the mean proportion of days pigs had diarrhoea with respect to 14 d after weaning [29]. Faecal samples from 8 focus pigs (median weight pigs) were collected per treatment, on d 4 and 11 after weaning, for subsequent volatile fatty acid (VFA) analysis and enumeration of specific bacterial groups by quantitative PCR (qPCR). Samples were stored at −20 °C until analyzed.

### 2.3. Blood Sampling

Blood samples were collected on d 4 and 11 from 2 pigs per pen (12 samples per treatment). Samples were collected via jugular vein puncture into either a lithium heparin or K_3_EDTA coated tube. Lithium heparin tubes were immediately placed on ice and K_3_EDTA tubes were kept cool. The heparin tube was centrifuged at 3000× *g* for 10 min at room temperature. Plasma was then collected and stored at −20 °C until analyzed for plasma urea nitrogen (PUN), total antioxidant content (TAC), and the acute phase proteins (APP) haptoglobin, albumin, and C-reactive protein (C-RP). Whole blood samples collected in K_3_EDTA tubes were subjected to blood cell count assessment on the same day.

### 2.4. Analytical Methods

Diet samples were analyzed for dry matter, gross energy, crude protein, crude fibre, neutral detergent fibre (NDF), acid detergent fibre (ADF), and zinc. Dry matter content was determined using AOAC official method 930.15 [30]. The N content was determined using combustion method 990.03 [30] and crude protein content was calculated as N content ×6.25. Crude fibre content was determined using AOAC official method 962.09 [30]. The NDF and ADF contents were determined using the AOAC official methods 925.10 [30]. Gross energy content was determined using a ballistic bomb calorimeter (SANYO Gallenkamp, Loughborough, UK). Zinc content was determined using inductively coupled atomic emission spectroscopy. The pH levels in the diets were assessed by preparing 1:9 *w*/*v* feed in water, which were then measured using a pH meter (ROSS Ultra^®^ pH/ATC Triode^®^, Thermo Fisher Scientific Inc., Beverly, MA, USA).

Faecal samples were analyzed for volatile fatty acids (VFA) content using gas chromatography. Samples were prepared as described by Kim, *et al.* [31], except that the thawed samples were diluted 1:2 (*w*/*v*), and expressed as the molar percentage of total VFA. The TAC was determined using the OxiselectTM Total Antioxidant Capacity (TAC) Assay Kit (Cell Biolabs, Inc., catalogue number #STA-360, San Diego, CA, USA). The PUN, haptoglobin and albumin contents were determined using a Beckman Coulter/Olympus Reagent Kit (OSR6134), an in-house method NTM-62 based on [32], and Randox Ranbut Reagent kit (RB1007), respectively. All kits and methods were performed on an Olympus AU400 Clinical Chemistry Analyser at the Department of Agriculture and Food, Animal Health Laboratories (South Perth, WA, Austrlia). The C-RP levels were determined by the use of a porcine ELISA kit (R & D Systems, catalogue #DY2648). Whole blood cell count was conducted using an automatic haematology analyser (ADIVA 2120, Bayer Healthcare, Siemens, Germany).

### 2.5. DNA Extraction and Quantitative Real Time Polymerase Chain Reaction

The QIAamp^®^ DNA Stool Mini Kit (QIAGEN GmBH, Hilden, Germany) was used to extract DNA from frozen faecal samples with DNA stored at −20 °C until qPCR was performed. Quantitative PCR was used to measure numbers of total *E. coli*, *E. coli* with F4 fimbria (the specific *E. coli* used for oral infection in this experiment), *Enterobacteriaceae*, *Lactobacillus* spp., and total bacteria by targeting 16S rRNA genes, the 16S–23S rRNA intergenic spacer region and the F4 fimbriae gene (Table 2). Primers and probes were synthesized by Biosearch Technologies (Novoto, CA, USA). TaqMan probes were labelled with 5′ carboxyfluorescein (FAM) on the 5′ end and Black Hole Quencher (BHQ-1) on the 3′ end (Table 2). Standard curves for each of the qPCRs were constructed from 10-fold serial dilutions of each DNA extracted using the DNeasy Blood and Tissue Kit (Qiagen, Venlo, Netherlands) from broth cultures of F4 *E. coli*, *E. coli*, *Salmonella typhimurium* and *Lactobacillus acidophilus*. Numbers of each bacterial species were enumerated using ISO 16649-2, ISO 21528-2 and ISO 15214 international standards.

**Table 2:** Primers and probes used for quantitative real time PCR.

Target	Primer/Probe	Reference	PCR Product Length (bp)
Total *E. coli*	Forward	Bartosch, *et al.* [33]	195
	Reverse		
	Probe	Designed by Y. Chen, EMAI (unpublished)	
*Enterobacteriaceae*	Forward	Bartosch, *et al.* [33]	195
	Reverse		
	Probe	Designed by Y. Chen, EMAI (unpublished)	
*Lactobacillus* spp*.*	Forward	Walter, *et al.* [34]	190
	Reverse	Modified from Heilig, *et al.* [35]	
	Probe	Modified from Delroisse, *et al.* [36]	
*E. coli* with F4 fimbria	Forward	Franklin, *et al.* [37]	764
	Reverse		
Total bacteria	Forward	Suzuki, *et al.* [38]	152
	Reverse		
	Probe		

The F4 *E. coli* qPCR was the only assay to use SYBR Green to detect DNA amplification, and all other assays used the TaqMan probe technology. Each TaqMan qPCR contained 1× AgPath-ID reaction buffer (Applied Biosystems, Foster City, CA, USA), 1 unit AgPath-ID Taq polymerase, 40 nM probe, and 200 nM of the forward and reverse primers. The qPCR amplification protocols for the total *E. coli*, *Enterobacteriaceae* and *Lactobacillus* spp. included an initial denaturation step of 95 °C for 10 min, then 40 cycles of 95 °C for 15 s and 65 °C for 45 s. A reduced annealing temperature was used (58 °C) for the total bacteria qPCR. The F4 *E. coli* qPCR contained 1× SensiMix SYBR Low-ROX reaction buffer including Taq polymerase (Bioline, London, UK) and 300 nM forward and reverse primers. Cycling conditions for the F4 *E. coli* qPCR were 95 °C for 10 min, then 40 cycles of 95 °C for 15 s, 65 °C for 30 s and 72 °C for 60 s. Samples were quantified on a ViiA7 PCR machine (Applied Biosystems, Foster City, CA, USA), and numbers of total *E. coli*, *Enterobacteriaceae*, *Lactobacillus* spp*.* and F4 *E. coli* were expressed as a percentage of total bacteria to account for differences in faecal water content.

### 2.6. Statistical Analyses

Statistical analysis of production data, faecal score, faecal ETEC excretion and DI was performed using one-way ANOVA in SPSS (Version 21, IBM Corporation, Armonk, NY, USA) with dietary treatment as the independent variable, and batch as a random factor (to account for a difference in start weight between batches of pigs). Plasma measures, faecal SCFA, faecal bacterial counts and ratios measured on d 4 and 11 were analysed by repeated-measures ANOVA. Faecal bacterial counts for total *E. coli*, F4 *E. coli* and *Enterobactericeae*, and faecal bacterial ratios for *Lactobacillus* spp.:*E. coli* and F4 *E. coli*:*Lactobacillus* spp., were not normally distributed and therefore were logarithmically transformed before analysis. Means were back-transformed and expressed as least-square means with 95% confidence intervals. Significant interaction means were separated using Tukey’s HSD test. Pen was used as the experimental unit for performance, faecal score, faecal ETEC excretion, and DI. Pig was used as the experimental unit for plasma measurements, blood cell counts, faecal VFA concentration, and bacterial enumeration by qPCR. Chi-squared analysis (SPSS; Version 21, IBM, Armonk, NY, USA) was used to compare the percentage of pigs having PWD between the different diets. Statistical significance was accepted at *p* < 0.05 and 0.05 < *p* < 0.10 was considered a trend.

## 3. Results

One animal was removed from the trial prior to the ETEC challenge due to ill thrift. The analysed diet composition did not vary significantly from calculated values. As expected, the Zn concentration was lower in the base and OACP diets (Table 1). The pH levels were as expected, with the ZnO diet having the highest pH and the OACP diet having the lowest pH (Table 1).

### 3.1. Incidence and Severity of PWD and Shedding of ETEC

Approximately 4% of pigs fed ZnO had PWD in the 3 weeks after weaning, which was lower than pigs fed OACP (29%, *p* = 0.024) or the base-fed pigs (25%, *p* = 0.047). There was no difference (*p* = 0.745) in PWD between pigs fed the OACP or base diets. This related to the DI, which was lower (*p* = 0.026) in pigs fed ZnO compared to pigs fed either the OACP or the base diet. There was an increase (*p* < 0.001) in the haemolytic *E. coli* score after infection, with no difference (*p* = 0.987) between treatments (Table 3).

**Table 3:** The effects of dietary treatment on post-weaning diarrhoea, the diarrhoea index, and the *E. coli* score before and after infection, in pigs experimentally infected with enterotoxigenic *E. coli* (ETEC).

Item	Treatment *			SEM	*p*-Value		
	Base	ZnO	OACP		D	T	D × T
% of pigs with PWD **^†^**	25 **^a^**	4 **^b^**	29 **^a^**				
Diarrhoea index (%) **^‡^**	5.06 **^a^**	0.62 **^b^**	6.25 **^a^**	1.526		0.026	
*E. coli* score **^§^**							
Days 0–3 (pre-infection)	0.08	<0.01	0.15	0.048	0.001	0.987	0.442
Days 5–11 (post-infection)	0.94	1.05	0.91				

* Base diet; ZnO: base + 3 g ZnO/kg; OACP: base + 1.5 g/kg of a mixture of an organic acid blend, cinnamaldehyde and a permeabilizing complex (Biotronic Top 3^®^, Biomin Australia Pty, Ltd., Carlingford, Australia); **^†^** PWD was defined as pigs having a faecal consistency score of 4; **^‡^** The mean proportion of days with diarrhoea with respect to 14 d after weaning; **^§^** Agar plates were scored from 0–5 according to number of streaked sections containing viable haemolytic *E. coli*, where 0 was no growth and 5 was growth out in the fifth section of the plate; PWD, Post weaning diarrhoea; SEM, standard error of the mean; D, day; T, treatment; **^a,b^** Means within a row with different superscripts are significantly different (*p* < 0.05).

### 3.2. Performance Data

Production performance of all pigs was sound, with the average daily gain (ADG) being in excess of 250 g/d in the first week after weaning. Pigs fed ZnO diet were heavier than pigs fed the base diet on d 14 (*p* = 0.024) and d 21 (*p* = 0.038), with pigs fed the OACP diet differing from neither (*p* > 0.05). The ADG was higher in pigs fed ZnO compared to the base diet-fed group for the first (*p* = 0.008) and the overall 3-week period after weaning (*p* = 0.013). Daily gain was higher for pigs fed ZnO or OACP compared to the base diet group in the second week (*p* = 0.011), however no differences were found for the third week after weaning (*p* = 0.421) (Table 4).

Pigs fed ZnO had a greater average daily feed intake (ADFI) compared to the base diet-fed pigs in week 1 (*p* = 0.007) and in the overall 3-week period after weaning (*p* = 0.004). Pigs fed ZnO or OACP had a greater (*p* = 0.006) ADFI compared to the base diet-fed group in week 2. For the 3 weeks after weaning, a trend (*p* = 0.057) was found for pigs fed ZnO to consume more food than the base diet group. There was no difference (*p* > 0.05) in feed conversion ratio between treatment groups (Table 4).

**Table 4:** The effects of feeding different diets on the performance of pigs experimentally infected with ETEC after weaning.

Item	Treatment *			SEM	*p*-Value
	Base	ZnO	OACP		
LW (kg)					
d 0	7.3	7.2	7.2	0.11	0.761
d 7	9.1	9.9	9.4	0.25	0.122
d 14	12.1 **^a^**	13.6 **^b^**	13.0 **^a,b^**	0.34	0.024
d 21	16.3 **^a^**	18.2 **^b^**	17.4 **^a,b^**	0.46	0.038
ADG (g/d)					
d 0–7	253 **^a^**	381 **^b^**	312 **^a,b^**	24.3	0.008
d 8–14	430 **^a^**	533 **^b^**	510 **^b^**	21.3	0.011
d 15–21	611	661	626	26.7	0.421
d 0–21	431 **^a^**	525 **^b^**	483 **^a,b^**	19.1	0.013
ADFI (g/d)					
d 0–7	336 **^a^**	454 **^b^**	390 **^a,b^**	21.8	0.007
d 8–14	543 **^a^**	725 **^b^**	668 **^b^**	33.6	0.006
d 15–21	835	942	871	28.9	0.057
d 0–21	571 **^a^**	707 **^b^**	643 **^a,b^**	23.6	0.004
FCR (g/g)					
d 0–7	1.34	1.18	1.26	0.059	0.218
d 8–14	1.25	1.36	1.30	0.045	0.282
d 15–21	1.37	1.43	1.39	0.039	0.515
d 0–21	1.32	1.34	1.33	0.029	0.847

* Base diet; ZnO: base + 3 g ZnO/kg; OACP: base + 1.5 g/kg of a mixture of an organic acid blend, cinnamaldehyde and a permeabilizing complex (Biotronic Top 3^®^, Biomin Australia Pty, Ltd., Carlingford, Australia); LW, live weight; ADG, average daily gain; ADFI, average daily feed intake; FCR, feed conversion ratio; SEM, standard error of the mean; **^a^**^,**b**^ Means within a row with different superscripts are significantly different (*p* < 0.05).

### 3.3. Quantitative Real Time PCR

The total number of bacteria in faecal samples ranged from 4.30 × 10^10^ to 5.69 × 10^11^, with an average of 2.52 × 10^7^, 1.57 × 10^9^, 2.69 × 10^8^ and 6.38 × 10^7^ for *Lactobacillus* spp., *Enterobacteriaceae*, total *E. coli* and F4 *E. coli* detected per gram of faeces, respectively. The percentage of total *E. coli* in faeces was not affected (*p* > 0.05) by treatment or day of sampling, and there was no significant interaction between day and treatment. The percentage of *E. coli* with F4 fimbriae was affected a day by treatment interaction (overall, *p* = 0.037), with more F4 *E. coli* found in pigs fed ZnO on d 11 (*p* = 0.011) compared to base diet-fed pigs. Similarly there was a day and treatment interaction (overall, *p* = 0.020) for *Enterobacteriaceae*, with pigs fed ZnO having more bacteria than pigs fed the base diet on d 11 only. There was no difference between pigs fed OACP and the base diet. There was a weak trend for an interaction between day and treatment for *Lactobacillus* spp*.* (*p* = 0.078) (Table 5).

**Table 5:** Selected bacterial counts (in percentage of total bacterial count) on day 4 and day 11 in pigs experimentally infected with ETEC and fed different diets after weaning.

Item	Treatment *			SEM	*p*-Value		
	Base Diet	ZnO	OACP		D	T	D × T
Total *E. coli* **^†^**							
d 4	2.3 (0.74–7.03)	1.1 (0.38–3.12)	3.9 (1.16–13.18)		0.715	0.405	0.386
d 11	1.4 (0.34–5.55)	2.8 (0.75–10.16)	4.2 (0.92–18.75)				
F4 *E. coli* **^†,‡^**							
d 4	0.017 (0.0022–0.13)	0.0073 (0.0011–0.049)	0.025 (0.0032–0.20)		<0.001	0.374	0.037
d 11	0.034 (0.0052–0.22)	1.05 (0.18–6.03)	0.30 (0.047–1.95)				
*Enterobacteriaceae* **^†^**							
d 4	34.2 (11.9–97.9)	21.0 (8.4–52.2)	22.2 (6.1–80.5)		0.169	0.595	0.020
d 11	6.4 (1.2–37.8)	46.1 (9.9–214.2)	14.6 (1.7–128.2)				
*Lactobacillus* spp*.*							
d 4	0.8	1.3	2.0	0.00	0.340	0.567	0.078
d 11	1.4	0.9	0.9				

* Base diet; ZnO: base + 3 g ZnO/kg; OACP: base + 1.5 g/kg of a mixture of an organic acid blend, cinnamaldehyde and a permeabilizing complex (Biotronic Top 3^®^, Biomin Australia Pty, Ltd., Carlingford, Australia); **^†^** Data that are not normally distributed were logarithmically transformed, then analysed by GML. Values were back-transformed and expressed as least square means with 95% confidence intervals (in brackets); **^‡^** Specific *E. coli* used for oral infection in the experiment; SEM, standard error of the mean; D, day; T, treatment.

There were some trends for interactions between treatment and day influencing several bacterial ratios including F4 *E. coli*:*E. coli* (*p* = 0.068), F4 *E. coli*:*Enterobacteriaceae* (*p* = 0.062), and *Lactobacillus* spp*.*:*Enterobacteriaceae* (*p* = 0.095). There were no day or treatment differences (*p* > 0.05) in the proportion of *Lactobacillus* spp*.*:*E. coli*, however the ratio of F4 *E. coli*:*Lactobacillus* spp*.* was influenced by a day by treatment interaction (*p* = 0.010). On d 11 only, ZnO-fed pigs and OACP-fed pigs had higher ratios compared to base diet-fed pigs (Table 6).

**Table 6:** Ratios of selected bacterial counts (expressed as a percentage) on day 4 and day 11 in pigs experimentally infected with ETEC and fed different diets after weaning.

Item	Treatment *			SEM	*p*-Value		
	Base	ZnO	OACP		D	T	D × T
F4 *E. coli* **^‡^**:*E. coli*							
d 4	40.2	3.5	7.6	8.20	0.321	0.963	0.068
d 11	6.0	51.7	38.0				
*E. coli*:*Enterobacteriaceae*							
d 4	15.0	237.1	10.0	0.29	0.592	0.048	0.705
d 11	13.3	111.4	16.7				
F4 *E. coli*:*Enterobacteriaceae*							
d 4	0.2	0.5	1.3	0.70	<0.001	0.083	0.062
d 11	1.1	8.2	6.8				
*Lactobacillus* spp*.*:*Enterobacteriaceae*							
d 4	5.3	21.6	35.0	10.90	0.394	0.705	0.095
d 11	64.7	6.4	27.6				
*Lactobacillus* spp*.*:*E. coli* **^†^**							
d 4	268	97.7	32.4		0.380	0.513	0.145
	(8.6–82.8)	(33.9–280.5)	(9.6–109.6)				
d 11	75.2	19.9	12.8				
	(13.0–435.5)	(3.9–102.8)	(1.9–85.3)				
F4 *E. coli*:*Lactobacillus* spp*.* **^†^**							
d 4	2.7	0.7	1.6		<0.001	0.450	0.010
	(0.4–20.3)	(0.1–4.5)	(0.2–12.1)				
d 11	3.3	191.7	47.3				
	(0.4–27.1)	(26.5–1386.8)	(5.7–392.6)				

* Base diet; ZnO: base + 3 g ZnO/kg; OACP: base + 1.5 g/kg of a mixture of an organic acid blend, cinnamaldehyde and a permeabilizing complex (Biotronic Top 3^®^, Biomin Australia Pty, Ltd., Carlingford, Australia); **^†^** Data not normally distributed were logarithmically transformed then analysed by GML. Values were back-transformed and expressed as least square means with 95% confidence intervals (in brackets); **^‡^** Specific *E. coli* used for oral infection in the experiment; SEM, standard error of the mean; D, day; T, treatment.

### 3.4. Blood Measurements

The levels of PUN decreased from d 4 to d 11 (*p* = 0.001), however there was no difference between treatments (*p* = 0.220). The TAC levels decreased from d 4 to 11 (*p* < 0.001), with no difference between treatments (*p* = 0.134). There was no difference over time or between treatments in levels of haptoglobin (*p* > 0.05). Albumin decreased from d 4 to d 11 (*p* = 0.016), however there was no difference between treatments (*p* = 0.752). The levels of C-RP increased from d 4 to d 11 (*p* = 0.003), with no difference between treatments (*p* = 0.267; Table 7).

**Table 7:** Plasma levels of plasma acute phase proteins, plasma urea nitrogen, and total antioxidant capacity on day 4 and day 11 in pigs experimentally infected with ETEC and fed different diets after weaning.

Item	Treatment *			SEM	*p*-Value		
	Base	ZnO	OACP		D	T	D × T
Haptoglobin (mg/mL)							
d 4	1.2	1.0	1.3	0.09	0.325	0.210	0.285
d 11	1.4	0.8	0.9				
Albumin (mmol/L)							
d 4	24.8	24.6	24.9	0.36	0.016	0.752	0.689
d 11	23.1	23.4	24.2				
C-RP (μg/mL)							
d 4	9.8	9.0	10.4	1.51	0.003	0.267	0.161
d 11	20.5	10.0	18.5				
PUN (mmol/L)							
d 4	3.3	3.1	2.7	0.11	<0.001	0.220	0.435
d 11	2.8	2.4	2.4				
TAC (μM)							
d 4	250.1	265.7	246.5	3.12	<0.001	0.134	0.659
d 11	213.9	224.8	218.3				

* Base diet; ZnO: base + 3 g ZnO/kg; OACP: base + 1.5 g/kg of a mixture of an organic acid blend, cinnamaldehyde and a permeabilizing complex (Biotronic Top 3^®^, Biomin Australia Pty, Ltd., Carlingford, Australia); C-RP, C-reactive protein; PUN, plasma urea nitrogen; TAC, total antioxidant capacity; SEM, standard error of the mean; D, day; T, treatment.

There was no difference between days or between treatments for levels of haemoglobin, proportions of lymphocytes and neutrophils, or the lymphocyte to neutrophil ratio (*p* > 0.05).

### 3.5. VFA Composition

There were no significant interactions between day and treatment for total VFA concentration or for molar ratios of VFA. Pigs fed the OACP diet had the highest levels of total VFA compared to pigs fed ZnO or the base diets (*p* = 0.013), however there was no difference between days (*p* = 0.475) (Table 8).

Only pigs fed the ZnO diet showed a change in the molar ratios of the VFA measured, with pigs fed ZnO having a lower (*p* = 0.003) molar ratio of valeric acid compared to pigs fed the base diet or the OACP diet. The molar ratio of acetic acid increased (*p* = 0.028) from d 4 to d 11 whereas the molar ratios of isovaleric and caproic acids both decreased from d 4 to d 11 (*p* = 0.024 and *p* = 0.017 respectively). There was a trend for a decrease in both isobutyric acid and valeric acid from d 4 to d 11 (*p* = 0.061 and *p* = 0.064, respectively) (Table 8).

**Table 8:** Volatile fatty acid composition on d 4 and d 11 in pigs experimentally infected with ETEC and fed different diets after weaning.

Item	Day after Weaning		Treatment *			SEM	*p*-Value		
	4	11	Base	ZnO	OACP		D	T	D × T
Total VFA (mmol/L)	50.4	55.1	49.7 **^a^**	49.1 **^a^**	61.1 **^b^**	1.57	0.475	0.013	0.832
Molar ratios									
Acetic acid (%)	51.9 **^a^**	54.5 **^b^**	54.9	54.1	50.7	0.84	0.028	0.139	0.279
Propionic acid (%)	22.3	21.8	23.2	21.4	22.0	0.82	0.586	0.673	0.756
Butyric acid (%)	14.7	15.9	13.4	15.9	15.6	0.73	0.660	0.416	0.117
Isobutyric acid (%)	2.5	1.9	2.4	2.0	2.3	0.18	0.061	0.650	0.946
Valeric acid (%)	4.4	3.6	4.9 **^a^**	2.9 **^b^**	4.9 **^a^**	0.24	0.064	0.003	0.578
Isovaleric acid (%)	3.9 **^a^**	2.6 **^b^**	3.6	3.0	3.5	0.33	0.024	0.683	0.929
Caproic acid (%)	1.2 **^a^**	0.6 **^b^**	1.0	0.7	1.1	0.10	0.017	0.344	0.943

* Base diet; ZnO: base + 3 g ZnO/kg; OACP: base + 1.5 g/kg of a mixture of an organic acid blend, cinnamaldehyde and a permeabilizing complex (Biotronic Top 3^®^, Biomin Australia Pty, Ltd., Carlingford, Australia); VFA, volatile fatty acids; SEM, standard error of the mean; D, day; T, treatment; **^a^**^,**b**^ Means within a main effect within a row with different superscripts are significantly different (*p* < 0.05).

## 4. Discussion

Data from this experiment showed that the incidence of PWD decreased only for the pigs supplemented with ZnO, as both the number of pigs with PWD and the DI were decreased. Despite there being a decrease in the incidence of PWD for the ZnO-treated group, the shedding of ETEC was similar for all groups. These data support other studies [39,40] suggesting that supplementation of ZnO suppresses PWD and increases growth performance, but that these effects are not necessarily related to a reduction in ETEC or faecal score but possibly to improved intestinal barrier and (or) epithelial immune functions. Roselli, *et al.* [41], for example, concluded that ZnO protects cells from ETEC by the inhibition of bacterial adhesion and internalization, preventing disruption of barrier integrity. This inhibition reduces levels of endotoxin production and decreases the severity of PWD. This may explain the decrease in DI but not in ETEC shedding for pigs fed ZnO in the present study. Furthermore, pigs may not express the receptors necessary for the *E. coli* to attach [42,43] and hence not display PWD whilst shedding ETEC. Other pigs may have receptors that are only weakly adhesive, and the presence of receptors also varies between individuals [43]. These factors will have a strong influence on the occurrence of PWD but not necessarily the faecal shedding of ETEC [43]. Unfortunately it was not possible to conduct genotype screening for F4 receptors in this experiment.

In the present study and in agreement with numerous other studies [44,45,46,47], ZnO addition to the diet increased growth rate and feed intake after weaning and reduced PWD. A plethora of reasons have been provided for the mechanism(s) whereby ZnO exerts positive effects in the post-weaning period (e.g., [4,48]). However, in the current study we were unable to determine the precise cause(s) for the improved production and reduction in PWD from the data collected. In addition, supplementation with OACP gave a statistically similar production performance as the ZnO-supplemented diet, however and aside from the second week after weaning, performance for the OACP-fed pigs was equivalent to the base-fed pigs. The OACP product contains a mixture of organic acids, cinnamaldehyde and a proprietary permeabilizing complex. An abundance of research has been conducted on organic acids and their effects on performance in weanling pigs, as summarized by Partanen and Mroz [13] and Mroz [49], among others. Meta-analyses of the data show that organic acids generally improve growth performance. However large variation exists due to factors such as form and type of the organic acid, inclusion level, production of intraluminal SCFA, differences in the amount of fermentable carbohydrate substrates in the diet for bacterial growth, colonization and activity leading to SCFA production, weaning age, presence or absence of bacterial receptors, and hygiene and welfare [49].

Essential oils (EO) such as cinnamaldehyde tend to have ambivalent effects on production and PWD in young pigs [22,50]. The EO are believed to exert positive effects through mechanisms including antibacterial actions, alteration of intestinal microbiota, increased digestibility and absorbance of nutrients, and antioxidative and immunomodulatory activities [51,52]. In relation to changes in microbiota, Muhl and Liebert [53] reported that the EO (carvacrol and thymol) did not elicit any change to specific groups of the intestinal microbiota. Jiang, *et al.* [54], however, found a decrease in counts of *Lactobacillus* spp. when supplementing with EO (thymol and cinnamaldehyde), whilst Castillo, *et al.* [55] found an increase in bacterial counts of *Lactobacillus* spp. when diets were supplemented with carvacrol and cinnamaldehyde. Li, *et al.* [56] found an increase in *Lactobacillus* spp. counts and a decrease in the *E. coli* count, and suggested this was due to lactobacilli being less sensitive to any antimicrobial effects of EO compared to potentially pathogenic bacteria such as *E. coli*.

In the current study and for the group of pigs supplemented with OACP, a decrease in *E. coli* counts and an increase in *Lactobacillus* spp. counts before and after ETEC infection was anticipated, as inclusion of organic acids is thought to decrease pH and increase proteolytic enzyme activity in the gastrointestinal tract (GIT), producing a more favourable environment for *Lactobacillus* spp. and suppressing *E. coli* populations [57]. This was not generally supported by our data. No significant decreases were found in the proportions of F4 *E. coli* with fimbriae (relative to total bacteria) and *Enterobacteriaceae* (relative to total bacteria), nor was an increase seen in the proportion of *Lactobacillus* spp. (relative to total bacteria), following infection with ETEC in pigs fed OACP. In contrast, pigs supplemented with ZnO showed increases in the proportions of F4 *E. coli* (relative to total bacteria) and *Enterobacteriaceae* (relative to total bacteria) following infection, demonstrating further that ZnO had no direct efficacy against F4 *E. coli*. Nevertheless Højberg, *et al.* [46] found a decrease in *Lactobacillus* spp. when piglets were supplemented with 2.5 g ZnO/kg, and suggested that the influence of ZnO on the GIT microbiota worked in similar ways as some AGP, reducing Gram-positive commensals rather than potentially pathogenic Gram-negative bacteria.

Measuring ratios of commensal to pathogenic bacteria to evaluate GIT health overcomes the potential need to account for differences in faecal dry weight [58]. Castillo, *et al.* [59] successfully used qPCR to quantify total bacteria, *Enterobacteriaceae* and *Lactobacillus* spp. numbers in the digestive fluid of pigs, finding that qPCR was more sensitive than traditional microbiological methods, but ratios of lactobacilli to enterobacteria were comparable between methods. In the current study, the trend for an increase in ratios of F4 *E. coli*:total *E. coli*, F4 *E. coli*:*Enterobacteriaceae*, and F4 *E. coli*:*Lactobacillus* spp*.* for the ZnO-fed pigs on d 11 only suggests that feeding ZnO exacerbated the presence of the F4 fimbriae subtype in the faeces relative to other bacterial populations. Only the ratio of total *E. coli:Enterobacteriaceae* was affected by the dietary treatment, this again being higher in ZnO-fed pigs. Numbers and ratios of *Enterobacteriaceae* including *E. coli* with F4 fimbriae were generally higher after infection in pigs fed ZnO compared to pigs fed the other diets, and were generally not reduced with OACP. These data are in general agreement with the recent work from Starke, *et al.* [60].

Given the sound growth rates achieved in this study, the decrease in PUN levels from d 4 to d 11 could be attributable to a decrease in microbial fermentation of nitrogenous compounds in the large intestine commensurate with enhanced protein digestion and amino acid absorption. Catabolism of amino acids by microbes produces NH_3_, which is converted to urea in the liver. The urea synthesized in the liver is either excreted as urine or diffused back into the caecum and combined into microbial nitrogen [61]. A decrease in urea could also be linked to more efficient utilization of dietary protein or decrease in protein breakdown [62]. As pigs overcome the post-weaning growth check, their protein requirements increase from 13.1 g/d (5–7 kg LW) to 23.1 g/d (7–11 kg LW) [27]. In the present study only one diet was fed for the entire 3-week trial period, thus as pigs became heavier there would be an increase in utilization (*i.e.*, less excess N) as the requirements increased.

The APP are proteins that are a part of the acute phase response, the early defence or innate immune system triggered by different stimuli such as trauma, infection, stress and inflammation [63]. The APP are divided into two categories, positive and negative, which increase or decrease during the acute phase response, respectively [64]. Haptoglobin, a positive APP, increases in concentration according to deteriorated health status, infection, inflammation or trauma [32]. No increase in haptoglobin levels was found in the present study. C-RP is another positive APP and would be expected to increase in levels over time after the challenge, which was observed in this study. It has been established that APP have different induction sensitivities, and hence some react to a lesser extent than others to the same infection/inflammation [65]. When measuring APP levels in pigs infected with ETEC, Houdijk, *et al.* [66] found that C-RP concentrations increased more than 10-fold, whilst haptoglobin concentrations only increased three-fold. Furthermore, the decrease in TAC concentration from d 4 to 11 suggests that the ETEC infection decreased the antioxidative capacity, however and as with the APP, no difference between treatments was observed in the present trial, indicating that they all elicited a similar inflammatory response to the *E. coli* infection.

Concurrently, the lack of any differences found in white blood cell counts, over time, in the present study confirm that the overall level of infection was most probably insufficient to cause a major health issue for the pigs. The lymphocyte to neutrophil ratio has been used previously as an indicator of the pigs’ responses to stress [67], and the lack of any difference in the current study supports this notion.

The increase in total VFA concentration in the OACP group compared to the base diet and ZnO groups was expected, as the OACP diet was supplemented with organic acids. The addition of organic acids aids in the acidification of the stomach, thus increasing proteolytic enzyme activity, which then may increase the digestibility of protein and amino acids [68]. It has also been found that an increase in VFA stimulates GIT epithelial cell proliferation and villous height, hence increasing surface area for absorption [69], and some studies have shown that increased production of VFA can reduce pathogenic bacterial numbers such as salmonella [70,71]. However, the lack of any improvement in growth rate and feed conversion ratio, or any significant decrease in *Enterobacteriaceae* numbers in the present study, indicates that the increase in total VFA levels in pigs fed OACP was unrelated to these measurements.

## 5. Conclusions

This experiment demonstrated that feeding a diet supplemented with a blend of organic acids (propionic, formic and acetic), cinnamaldehyde and a permeabilizing complex, in comparison to a diet with ZnO or a base diet, did not decrease the incidence of PWD and did not increase growth performance of pigs experimentally infected with ETEC. Numbers and ratios of *Enterobacteriaceae*, including *E. coli* with F4 fimbriae, were generally higher after infection in pigs fed ZnO compared to pigs fed the other diets, and were not reduced with OACP. Nevertheless, pigs in the present study performed very well and the overall level of infection challenge was most likely low, hence testing the additives under more challenging commercial conditions may be warranted.
